# A comparative analysis of three vector-borne diseases across Australia using seasonal and meteorological models

**DOI:** 10.1038/srep40186

**Published:** 2017-01-10

**Authors:** Margaret D. Stratton, Hanna Y. Ehrlich, Siobhan M. Mor, Elena N. Naumova

**Affiliations:** 1 Tufts University Initiative for Forecasting and Modeling of Infectious Diseases (InForMID), 196 Boston Ave, Medford, MA 02155, USA; 2 School of Life and Environmental Sciences and Marie Bashir Institute of Infectious Diseases and Biosecurity, The University of Sydney, Australia; 3 Friedman School of Nutrition Science and Policy, Tufts University, 150 Harrison Avenue, Boston, MA 02111, USA

## Abstract

Ross River virus (RRV), Barmah Forest virus (BFV), and dengue are three common mosquito-borne diseases in Australia that display notable seasonal patterns. Although all three diseases have been modeled on localized scales, no previous study has used harmonic models to compare seasonality of mosquito-borne diseases on a continent-wide scale. We fit Poisson harmonic regression models to surveillance data on RRV, BFV, and dengue (from 1993, 1995 and 1991, respectively, through 2015) incorporating seasonal, trend, and climate (temperature and rainfall) parameters. The models captured an average of 50–65% variability of the data. Disease incidence for all three diseases generally peaked in January or February, but peak timing was most variable for dengue. The most significant predictor parameters were trend and inter-annual periodicity for BFV, intra-annual periodicity for RRV, and trend for dengue. We found that a Temperature Suitability Index (TSI), designed to reclassify climate data relative to optimal conditions for vector establishment, could be applied to this context. Finally, we extrapolated our models to estimate the impact of a false-positive BFV epidemic in 2013. Creating these models and comparing variations in periodicities may provide insight into historical outbreaks as well as future patterns of mosquito-borne diseases.

Seasonal patterns of disease, understood as periods of high and low disease incidence that are consistent across years, were recognized as early as 380 BCE^1^. Seasonality in vector-borne diseases (VBDs), in particular, is a well-known epidemiological phenomenon, in part because vectors are so highly influenced by meteorological and environmental conditions^2^. Understanding the intricacies of seasonal fluctuations in VBDs is critical in the optimization of forecasting and control efforts^2^,^3^.

Temperature and rainfall are frequently used in models to predict incidence of VBDs. In addition, a Temperature Suitability Index (TSI) has been used in previous studies to estimate mosquito abundance, another proxy for disease incidence^4^,^5^. The TSI method involves reclassifying weather data based on its suitability relative to optimal climate conditions for vector establishment and may be useful in assessing the impact of weather on disease for macro-geographic scales.

In Australia, Ross River virus (RRV), Barmah Forest virus (BFV), and dengue are three of the most common and clinically important VBDs. All display notable seasonal patterns and are transmitted by mosquitos. RRV and BFV contribute the largest annual disease burden and are endemic to Australia, whereas dengue exhibits periodic epidemic activity currently limited to the northeast corner of Australia^6^,^7^,^8^. RRV and BFV have several mosquito genera implicated in their transmission while dengue has only one local vector species, *Aedes aegypti*. Such distinctions may influence or explain underlying differences in the spatio-temporal patterns of these infections. Temperature and rainfall have repeatedly been found to be significantly associated with all three diseases in Australia, although the exact relationships are disputed^6^,^7^,^8^,^9^,^10^.

The national disease system does not routinely collect or report the country of acquisition for BFV or RRV cases^11^,^12^, yet for dengue the importation is significant^13^. Because BFV only occurs in Australia, there is no significant risk of importation of the virus from overseas^6^. Although RRV is endemic to Australia, there has been occasional epidemic activity in Papua New Guinea and the South Pacific, and importation into Australia from travelers to these regions is possible^14^. For example, New Zealand has reported infrequent importations of RRV from Fiji^14^. The risk of RRV acquisition is likely primarily local, given the large case burden of RRV throughout Australia and its short period of viremia in humans, as well as the fact that its main vertebrate hosts are believed to be non-migratory native macropods common to Australia, such as kangaroos and wallabies^15^. Nonetheless, RRV is found in dozens of vector and mammal host species and, given its recent spread from Australia into the South Pacific as well as Australians’ increasing overseas travel patterns, the virus may continue to expand to other tropical regions. Hence, improved data collection on the region of acquisition and relevant data analysis should allow better capturing seasonality and geographic spread.

Prior to this study, a comparison of the seasonal patterns of RRV, BFV, and dengue could only be achieved through comparison of models reported in existing literature. These models often use different predictor variables or disease data aggregated to different time and geographic units^4^,^6^,^7^,^8^,^10^,^16^. Here we provide a standardized evaluation and comparison of the temporal patterns of RRV, BFV, and dengue across continental Australia. We adapted an analytic framework by Naumova *et al*. that takes a parametric modeling approach to seasonality using Poisson linear regression and incorporates harmonic wave functions and meteorological data as the predictor variable^17^,^18^,^19^. Modern surveillance systems are primed to expand the conventional tools available to better define seasonal patterns and consistently estimate seasonal peak timing. The time series modeling of surveillance data is quickly gaining scientific rigor and software support and many options for statistical modeling exist; yet, they share potential deficiencies with respect to implementation and interpretation^2^. Thus, we chose to use linear regression instead of other popular modeling approaches (such as SARIMA models or machine learning) because of transparency in interpretation, reproducibility of results and the ease of interpretation.

For each disease and location, we assessed the relative contributions of trend, intra-annual seasonality, temperature and rainfall, time-lag, and inter-annual periodicity to the overall temporal pattern. We also examined the temporal patterns in dengue cases acquired overseas. We demonstrated the utility of such models by (1) assessing severity of historical dengue outbreaks, (2) extrapolating historical disease incidence between 1980–1990, (3) comparing the patterns of local and imported dengue case rates, and (4) estimating BFV incidence from 2013–2015, during which time the incidence of BFV may have been inflated due to diagnostic ambiguities^20^. As this study shows, secondary analyses of publicly available data, even data that is highly aggregated both spatially and temporally, can offer new perspectives and insights.

## Data and Methods

### Data Abstraction

We conducted this analysis using publicly-available disease incidence rates aggregated monthly from each state or territory by the Australian National Notifiable Disease Surveillance System (NNDSS)^21^. The states/territories in this study include: Western Australia (WA), Northern Territory (NT), Queensland (QLD), New South Wales (NSW), South Australia (SA), Victoria (VIC), and Tasmania (TAS). Australian Capital Territory (ACT) is a small territory surrounded on all sides by NSW; for the purposes of this study, case counts from NSW and ACT were aggregated together and this region referred to as NSW/ACT. The NNDSS also provided monthly disease incidence rates for each state and for Australia as a whole (Table S4).

The NNDSS provided case counts and disease incidence rates beginning in January 1991, 1993, and 1995 for dengue, RRV, and BFV, respectively. Data used in this study terminated in May 2015. However, beginning October 2012, Australia experienced a BFV “epidemic” that was later attributed primarily to errors in testing and subsequently an over-reporting of false positives^22^. Therefore, analysis of BFV data was limited to the period before the onset of the “epidemic” so as to capture more realistic data for our models. The only missing data points in the disease incidence data are for dengue in ACT from January 1991 to December 1992. However, in the 40 months of data following this missing period, no cases of dengue were reported in ACT. Missing points were therefore considered to be months with zero incidence of dengue. In order to determine the contribution of imported cases we compiled the annual reports from the Australian Department of Health^23^ (Table S1), tracked the progress of the surveillance system in improving the recording of potential exposure location (Table S2) and compiled the reporting of cases acquired overseas by country of acquisition (Table S3).

Data on total monthly rainfall and average monthly minimum and maximum temperature for the period January 1980 to May 2015 were obtained from Australia’s Bureau of Meteorology which records, and makes public, measurements from individual weather stations^24^. We selected data from weather stations in each of the 110 cities defined as Significant Urban Areas (SUAs) by the 2011 census from the Australian Bureau of Statistics. SUAs are standard geographic units consisting of regions with a population >10,000, among other criteria^25^. Australia’s population, and the number of SUAs, varies greatly between states, hence the number of weather stations used for weather abstraction also varied between states. For example, 36 stations were used for NSW/ACT whereas only two were used for NT. Eight cities were excluded because weather data were unavailable. In order to maintain consistency with our disease data, we averaged the values from all weather stations in each state by month and weighted these averages by population size. The monthly weather data was 97.9% complete for the study period. Missing values were not included in the average and the weights were appropriately adjusted. The resulting monthly state averages had no missing values.

### Seasonal and Meteorological Modeling of VBD Incidence

Using the statistical software R (version 3.2.3), we created observational time series plots and calendar plots to identify seasonality in the disease data. We then created a series of Poisson linear models for each disease and state, consistent with methods used by other researchers when modeling one or more seasonally-affected diseases^6^,^17^,^26^,^27^,^28^. Table 1 shows the progression of equations. The models were built from a basic regression equation with only linear trend (step 1) and expanded upon by adding a seasonality component of one peak per year (i.e. with a 12-month period) (step 2). Finally, average minimum and maximum temperature and total rainfall were added (step 3) to create Model A.

### Model Improvements: Lags, Additional Periodicities, and Temperature Suitability Index

The model was expanded further to include a lag on the weather data. This was to account for potential delays in the time in which weather affects mosquito abundance, which in turn affects disease incidence. To determine the amount of lag, 13 intermediate models were created for each disease and state using monthly lags ranging from 0 to 12 months. The lag corresponding to the model with the highest percent variability explained was used in subsequent modeling for that specific disease and state (Table 2, Step 1). The model was then expanded further to incorporate three more periodic components with periods greater than 12 months to allow for years with high disease incidence to be followed by years with lower disease incidence (Step 2). These periods were chosen using a Fourier transformation analysis over the length of the study to determine the periodic functions that best describe the raw disease rate data^29^. For each disease, the three periods which best described disease incidence across all locations (rounded to the nearest year) were selected for Model B. The models’ coefficient values can be found in Table S5.

Weather data was included in the models as a way of estimating mosquito abundance. However, Models A and B do not explicitly incorporate optimal temperature ranges for the mosquito vector species. Thus, Model C was developed utilizing the Temperature Suitability Index (TSI) instead of raw temperature data. The TSI index, described by De Wet *et al*., converts temperature to a value between 0 and 10 (0 being temperatures at which the vector species cannot survive, and 10 being optimal temperatures)^5^. We utilized the threshold values provided by De Wet *et al*. for *Culex annulirostris*, a common vector for both BFV and RRV in Australia that inhabits a wide variety of environments, for the TSI analyses of BFV and RRV^30^. We determined threshold values for *Aedes aegypti* based on larval survival rates at various field sites in Australia for our dengue analysis; *Ae. aegypti* is the only dengue vector in Australia and thrives in urban areas^31^. Minimum, maximum, and optimal condition thresholds were 16 C, 40 C, and 25 C to 37 C, respectively, for dengue and 9.7 C, 37.5 C, and 17.5 C to 27 C, respectively, for BFV and RRV^32^,^33^,^34^ (Figure S1). Average maximum and minimum temperatures were transformed using the TSI index and included in the model (Table 3, Step 2).

Next, since rainfall affects mosquito abundance differently depending on the season, the values for total rainfall were separated based on temperature into rain during cold, warm, and hot periods (step 3)^35^. The cutoffs to determine cold and hot were the same as the extreme minimum and maximum temperature values used in the calculation of TSI. Therefore, the number of rainfall variables in the final models increased from one to three.

### Estimations of model fit

The percent of variability explained by each model was calculated from the null and residual deviance and was used to compare how well each model fit the data for each disease and state. We were particularly interested in assessing the contribution of trend, seasonality, rainfall/temperature (with and without lag) and inter-annual periodicity to the temporal patterns. This was accomplished by measuring the change in variability explained between the nested models (shown in Tables 1 and 2) by comparing the difference in the overall variability and regression-based variability expressed by the per cent variability explained. Therefore, we assessed percent variability explained by each parameter independently, in addition to overall model fit. This approach is sufficient for comparing nested models applied to the same set of data to review the relative fit improvement. In order to get insight on the quality of fit for extreme values associated with outbreaks, the models were also assessed by measuring their residual skew and kurtosis^36^. Based on the departure of skewness and kurtosis estimates from expected values of 0 and 3, respectively, we judged the model’s tendency to over- or under-predict.

### Estimations of outbreak magnitude

We examined our models’ ability to estimate outbreak magnitude with a case study on dengue in QLD, the only state where domestic epidemic spread is a risk due to establishment of the vector *Aedes aegypti*^37^. Vector abundance and local transmission risk is highly influenced by weather conditions, and because our models utilize weather as a predictor variable, QLD is the most suitable state to evaluate the usability of our models^26^,^27^,^32^,^38^.

In order to demonstrate the use of the models to estimate outbreak magnitude, we considered the five largest and best-documented outbreaks in the period of 1991(the year that dengue became nationally notifiable) to 2009^39^,^40^. The selected five outbreaks primarily affected the city centers of Cairns and Townsville. Because we abstracted and weighted weather data by city size, we believe the proposed models likely captured local climate conditions for these outbreaks. We defined the period and duration for each outbreak using published articles that included specific temporal details on the respective outbreak.

First, we estimated the expected monthly values using model parameters and weather data within the specified period of each outbreak. We then determined excess values by subtracting expected monthly disease counts from observed counts and summing the residuals for each reported outbreak. Finally, we calculated the severity index as the ratio of observed versus expected total counts.

### Model Extrapolations

As noted previously, disease data for BFV used in this study was limited to the period January 1995 to October 2012, due to a false “epidemic” that commenced subsequently. Using the temperature and rainfall data to estimate disease incidence according to the models’ equations, we extrapolated the models for BFV forwards to May 2015 to predict incidence during this period without the influence of potentially false case data. In addition, we used weather data from 1980 to 1990 to extrapolate the models backwards for each disease to estimate the historical disease burden in each state and the total for all of Australia.

### Handling Imported Cases

Unlike BFV and RRV, dengue is found throughout the tropics. Cases of dengue are commonly imported into Australia from abroad and the data obtained from the NNDSS does not distinguish between locally-acquired and imported cases. While it is still important to model total notification rates regardless of place of acquisition in order to predict disease burden, the presence of many imported cases could obscure seasonal patterns and reduce the significance of weather parameters in the models. Therefore, we created additional models using data from the Australian Department of Health which measured in-country acquisition of dengue throughout Australia for July 2007 to June 2013^41^. The count data was converted to a rate using quarterly population estimates from the Australian Bureau of Statistics^42^. Following the procedure shown in Tables 1 and 2, a model of locally-acquired dengue was built and was compared to a model containing only imported cases.

## Results

**Figure 1:**
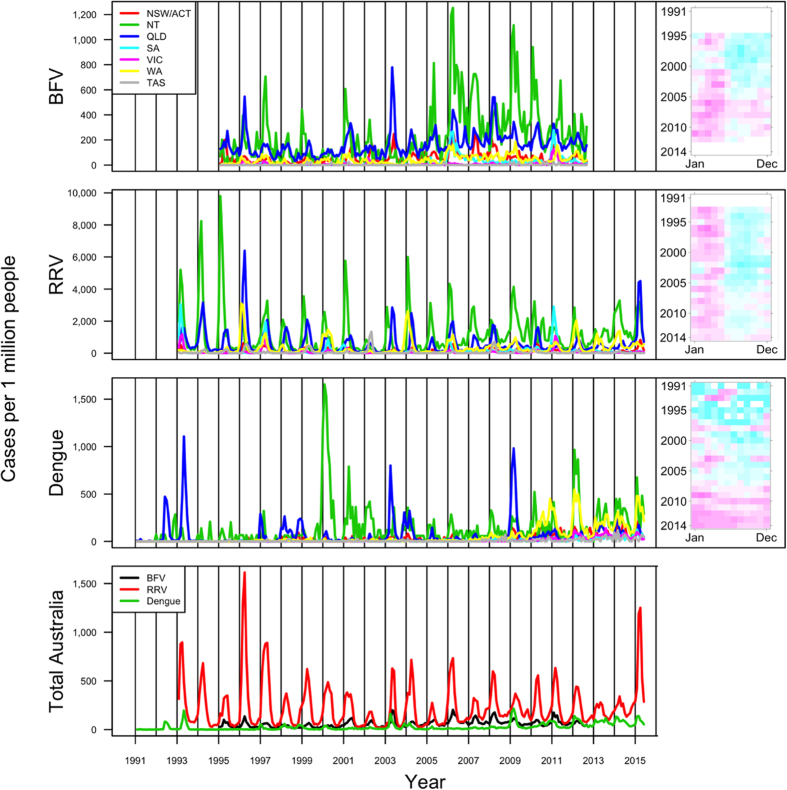
Disease incidence over time, measured in number of cases per million people per month. The first three graphs, separated by disease, compare disease incidence between states. Calendar plots for disease incidence across Australia are also shown (pink and blue represent months with high and low disease incidence, respectively). The fourth graph compares total disease incidence across Australia for each disease. Disease data is aggregated by month in the range Jan 1995–Oct 2012 for BFV, Jan 1993–May 2015 for RRV, and Jan 1991–May 2015 for dengue.

Figure 1 depicts raw surveillance data for each disease and location. As visualized by the contrasted pink and blue areas in the calendar plots to the right of the time series, disease incidence for BFV and RRV showed distinct and consistent seasonal patterns, with the highest disease burden typically occurring during the first five months of the calendar year. The seasonal pattern for dengue, in contrast, was less visible and was masked by the predominant increasing trend.

**Figure 2:**
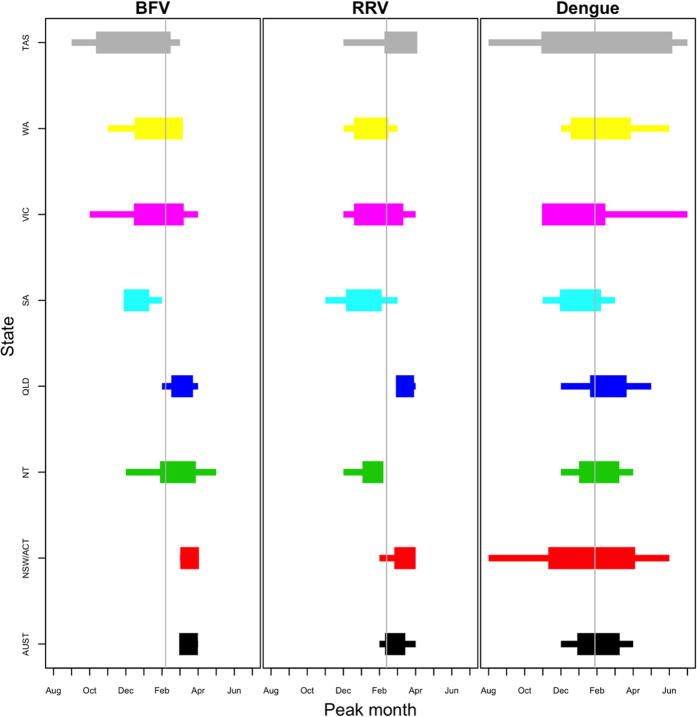
Mean peak timing for Model B for each state and disease. The boxes represent one standard deviation away from the mean peak time and the lines represent the full range of peak times seen in the model. Disease data is aggregated by month in the range Jan 1995–Oct 2012 for BFV, Jan 1993–May 2015 for RRV, and Jan 1991–May 2015 for dengue.

This difference in the strength of seasonality between the diseases is further exemplified in Fig. 2 which depicts the range of peak times for each model. The variability in peak times is highlighted by showing both the full range of peak times for a model and the range of peak times within one standard deviation of the mean. The dengue models have larger standard deviations and wider ranges of peak timings than the BFV and RRV models which reveals that the peak timing for dengue was more variable than the peak timings for BFV and RRV. The states with the most consistent peak timing were NSW/ACT, QLD, and SA for BFV and NSW/ACT, QLD, and NT for RRV. However, the models for disease counts aggregated across all of Australia tend to have more consistent peak timings than the models for individual states. Figure 2 also highlights the difference in peak timings between states: VIC, WA, and TAS tended to peak earlier than the other states.

**Figure 3:**
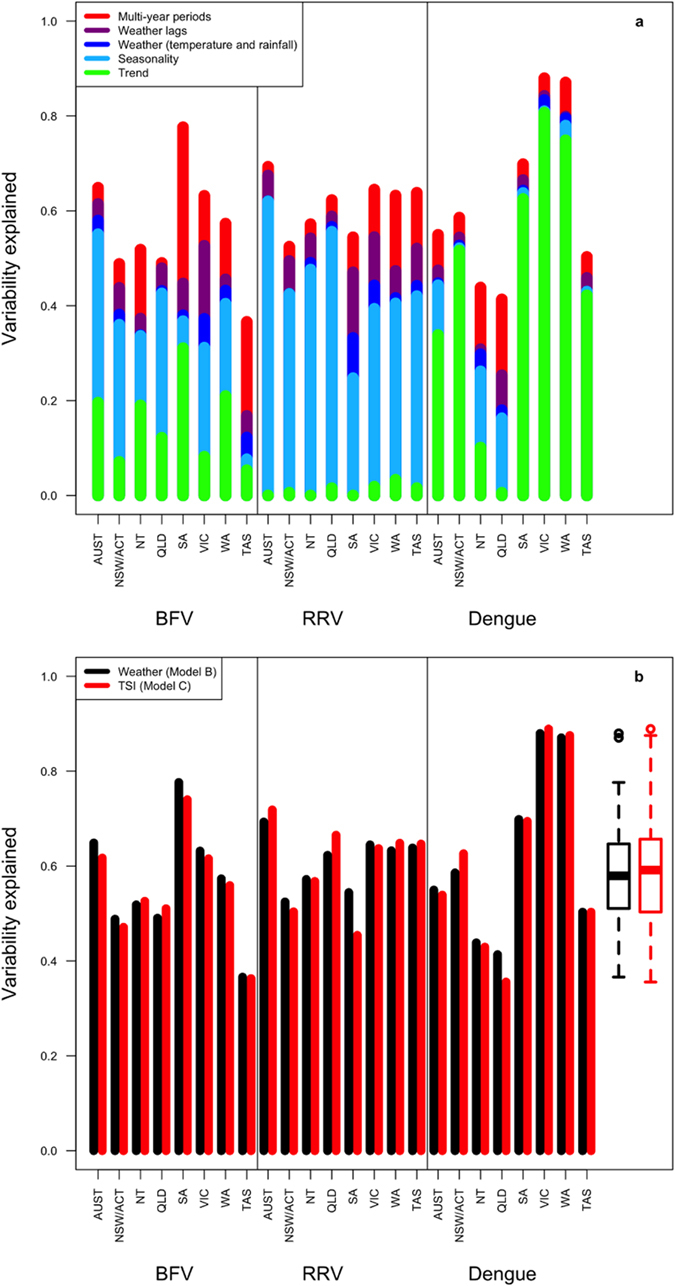
(**a**) Variability explained for Model B across each state and disease (Model B contains linear trend, seasonality, rainfall and temperature with lag, and multi-year periodicity). (**b**) Model B (using raw weather data) vs. Model C (using TSI and rainfall separated into temperature classes). The boxplots compare the average variability explained across all states and disease for Models B and C.

Our models attempted to understand the potential underlying factors and characteristics that contribute to the observed seasonal patterns. Figure 3a shows the variability explained by each model parameter for each disease and state. Overall, the average variation explained was just under 60%. The linear trend parameter (green bars) contributed the most to the models for dengue and was also important for BFV, while RRV appeared to have little to no change in trend over the study’s time period. In contrast, the seasonality parameter explained the highest proportion of variability for RRV (light blue bars). In general, adding weather data to the models (dark blue bars) did not substantially improve model fit. However, incorporation of lagged weather data improved model fit for BFV and RRV (purple bars). Addition of inter-annual periodicities to the models (red bars) had a profound effect for some states and diseases (such as BFV for SA). Variation explained for each disease and location was not significantly different between Model B (using raw weather data) and Model C (using TSI) as shown by the boxplots in Fig. 3b.

**Figure 4:**
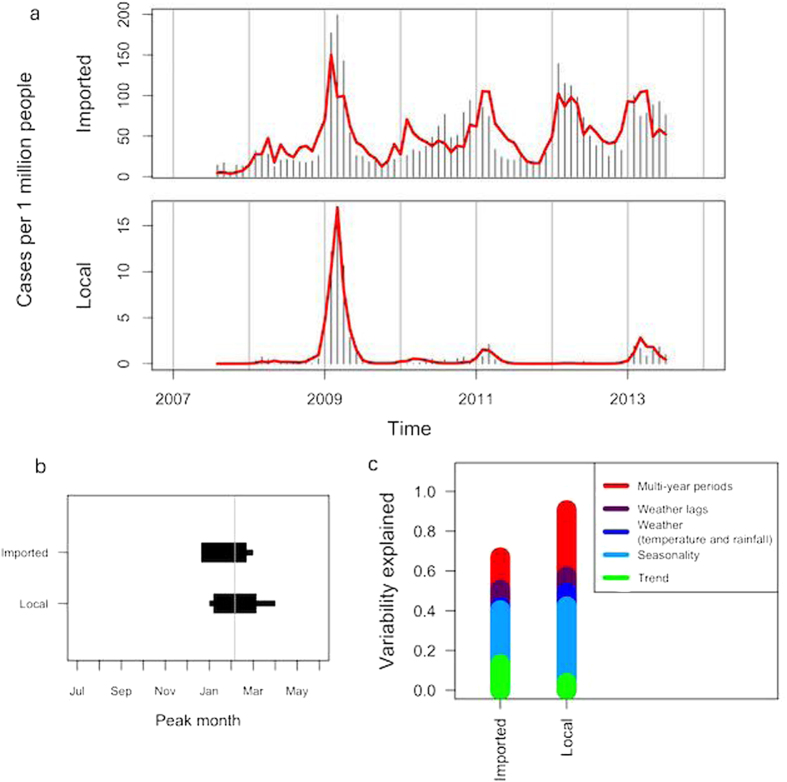
Comparison of dengue models built from imported and locally-acquired cases. (**a**) Prediction of disease incidence for Australia from Model B (red) and actual disease incidence for Australia (black); (**b**) Average peak timing (boxes represent one standard deviation from the mean and the lines represent the full range of peak times seen in the model; (**c**) A comparison of the variability explained for Model B for both dengue models.

The models’ residuals had average skewness and kurtosis values of 2.6 and 17.4 for BFV, 1.3 and 12.5 for RRV, and 4.7 and 36.6 for dengue (Table S9). Additionally, the plots of the models’ residual skew reveals that the models’ estimates tended to be lower than actual case counts (Figure S2) and the models did not sufficiently fit the data’s peaks. When considering the five largest outbreaks of dengue in QLD - the only state where local transmission occurs - from 1991–2009, our models under-estimated the first outbreak in 1992–1993, slightly over-estimated the subsequent outbreak in 1996–1997, and under-estimated the subsequent three outbreaks by 35–48% (Table S8). The severity index illustrates that the outbreak of 1992–3 was 2.7 times higher than can be explained by seasonal environmental variations. Similarly, the outbreaks of 1997–9, 2003–4, and 2008–9 were 1.5–1.9 times higher than can be explained by seasonal variation alone. In contrast, the model built using only locally-acquired cases of dengue, while having a higher variability explained than the models using imported cases, over-estimated the 2008–9 outbreak and the 2013 peak (Fig. 4). Both local and imported cases peak around January–February, and the standard deviation for peak timing for each model is approximately one month (Fig. 4b).

**Figure 5:**
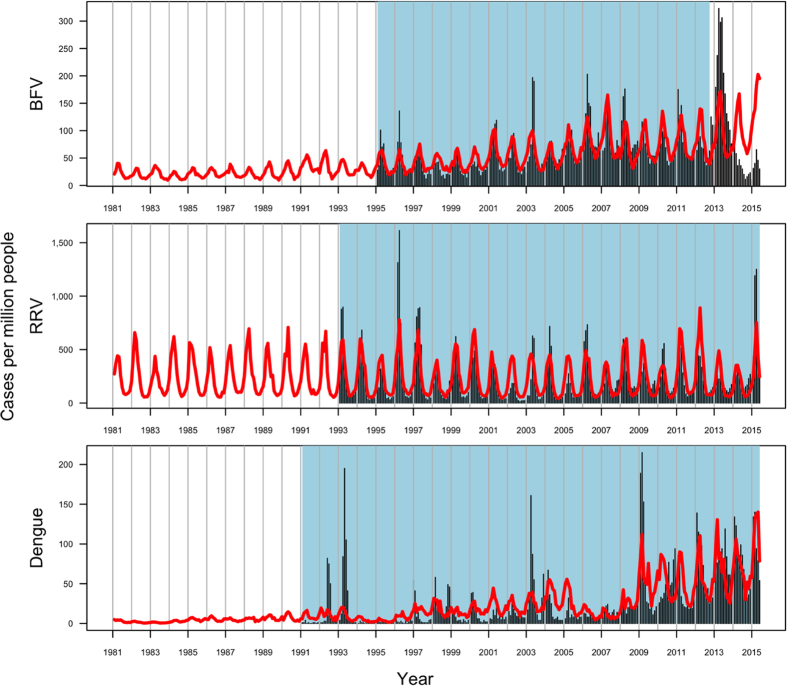
Prediction of disease incidence for Australia from Model B (red) and actual disease incidence for Australia (black). Light blue regions indicate the months of disease data used to build the model, all other months show extrapolations from the models.

Finally, we extrapolated Model B backward to January 1981 for each disease and forward to May 2015 for BFV on total Australian disease rates (Fig. 5). This revealed the models’ predictions for historical disease incidence, and potentially for actual disease incidence during the false epidemic of BFV in 2013–2014. When the BFV model was extrapolated forward, it predicted fewer cases than were reported, but still predicted a higher peak in disease incidence than average. In the original observation range, highlighted in blue, models consistently under-predicted disease counts for both RRV and BFV but performed much more variably for dengue over the study period.

## Discussion

This is the first study to present a comparative time series analysis for the major vector-borne diseases in Australia. Using parametric harmonic regression models, we were able to estimate the peak timing of disease incidence between each location and disease, revealing that RRV and BFV peaked in January or February, with SA tending to peak earlier and QLD and NSW/ACT tending to peak later. Dengue’s peak activity was more variable but generally occurred between November and March. Further, our findings highlight the need for careful consideration of trend, seasonality, temperature and rainfall, time-lag, and inter-annual periodicities for each disease, and stress that the relative importance of these parameters differs by disease and in some cases, location. Models based on these covariates explained 38% to 89% of variation in disease data, with the majority explaining 50% to 65% of the data.

We assessed the impact of each individual parameter in our models - for each disease and at each location–to better understand differences in the impact of covariates (Fig. 3). Dengue exhibited the greatest linear trend for the three diseases. However, when comparing dengue between locations, trend had the least influence in QLD and NT. The vector for dengue, *Ae. aegypti*, is currently limited to QLD, yet there have been numerous recent incursions of *Ae. aegypti* into towns in the NT^8^,^43^. Predictably, the addition of seasonality and lagged weather data therefore played a more significant role in the models for QLD and NT compared to other states where *Ae. aegypti* is not found. On the other hand, in all other states, trend played a much more significant role. This pattern is unlikely to be due to a change in the virus or mosquito distribution because cases of dengue in these states are imported rather than locally acquired. We suspect the increasing trend for dengue reflects the long-term growth in Australia’s travel sector, with increasing numbers of Australians traveling to locations in southeast Asia where dengue is endemic^13^,^44^. Furthermore, the minor influence of seasonal and weather patterns displayed for dengue in these locations may represent the seasons during which residents are most likely to travel. This explanation is supported by the fact that the peak for dengue in QLD and NT tended to occur in February (similar to that of RRV and BFV), but occurred earlier in the season, around November, for SA, NSW/ACT, and VIC, a pattern which again may reflect underlying seasonal travel patterns.

In contrast to dengue, trend for RRV was almost nonexistent, and the most important addition to the models was seasonality (Fig. 3). The SA and VIC models were most improved by the addition of weather data and lags. BFV showed an increasing trend, although less pronounced than dengue, and followed a seasonal pattern, though not as strongly as RRV (Fig. 3). The most striking finding for BFV is the increase in variability explained for SA when longer periods (24, 60, and 96 months) were added to the model. This could reflect a larger multi-year fluctuation in mosquito population sizes or temporary immunity following outbreaks.

The potential utility of these models is exemplified by our extrapolation of the models to estimate predicted case counts during the 2012–2013 outbreak of BFV. Officials attribute this outbreak primarily to an increase in false positive BFV diagnoses. The cross-reactivity between RRV and BFV in serological testing may have resulted in many dual notifications of the diseases during the outbreak, with estimations of up to 89% of cases testing falsely positive in the case of NT^20^. Following the outbreak, the case definition of BFV was altered to ensure a more specific diagnosis^20^. Our model prediction was lower than the reported incidence during the 2012–2013 outbreak, which supports the belief that the epidemic consisted of many false positives. Yet the prediction for BFV for the following two years, 2014 and 2015, was significantly higher than observed rates. On one hand, extrapolation of our model still suggests a nearly unprecedented peak in disease incidence in 2013, and therefore health officials may still need to be cautious in their current response to BFV since it is possible that a legitimate outbreak may have occurred during that time period. However, because our model over-predicted the rates for the subsequent years, the change in surveillance measures is likely the primary contributor to the dramatic fluctuations in observed BFV rates in recent years.

Surveillance measures can also help explain the magnitude of historical outbreaks. For example, the dengue outbreak of 1992–3, the largest in northern QLD in over a decade, had the highest Severity Index. As there was no organized control program at its onset, it is not surprising that an importation of the virus led to an explosive epidemic^45^. The Dengue Fever Management Plan (DFMP) was created following the outbreak, and this program alongside selective indoor residual spraying drastically helped curb the spread of the 1996–7 outbreak. These preventative efforts might explain our over-estimation of that epidemic^45^. Yet, as previously noted, the last decade has seen an upwards trend in dengue notifications and outbreaks^40^. Our models suggest that there are additional factors beyond seasonality or even surveillance measures, such as social trends and epidemiological factors, that contributed to the magnitude of outbreaks. For instance, the 2008–9 outbreak involved a strain of dengue with an unusually rapid transmission cycle and also reflected the larger trend of increased overseas travel to dengue-endemic countries. These factors may explain the high severity index of the 2008–9 outbreak, the largest in fifty years in QLD^40^,^46^,^47^. Although the models cannot be used as precise estimates of an outbreak’s scale, they can shed light on the magnitude of an outbreak and highlight potential changes in disease surveillance.

When averaging environmental data across large regions, a substantial fraction of climate variability within those regions may be lost. This is one of the difficulties of using environmental parameters to predict state-level disease incidence. Whelan *et al*. illustrate the challenges of using rainfall as a predictor across even relatively small distances^48^. In that study, rainfall exceeding 100 mm was found to be a good predictor of increased RRV risk in Alice Springs but not Tennant Creek, two cities within 500 km of each other. Because our weather data was aggregated by month and averaged across the entire state, we lost significant local climate variation and the results reflect the relationships at a coarse geographical scale. States with a greater number of SUAs contained more weather data observations and therefore likely estimated the weather across the state better than states with fewer SUAs. Further, we made the assumption that most disease cases originate in areas of high human density (and hence the weather data was weighted by city population size). While the vector for dengue, *Ae. aegypti*, is highly adapted to urban environments and tends to thrive in man-made urban settings, the vectors for BFV and RRV are found in a much larger variety of habitats and may require a different method of weather abstraction^30^,^49^. One potential alternative to the sparse ground monitoring network would be to use remotely-sensed indicators as proxies to environmental conditions and vector habitats. While the availability of historical satellite imagery might be limited, the availability and reliability of remote sensing at refined spatial scales is rapidly increasing.

This study attempted to combat some limitations of habitat variability through the use of a TSI as a means to recalibrate weather data based on more generalized trends in vector behavior. Because the difference between Models B and C were not significant (Fig. 3b), we can conclude that the decision to use actual weather data versus re-calculated weather data based on a rating system does not greatly change model fit. The TSI greatly simplifies model interpretation. Any temperature below the minimum or above the maximum gets mapped to zero and any temperature within the optimum range gets mapped to 10, so any variability within these ranges is neglected. This means that having high accuracy within specific temperature ranges (between the extreme and optimum values) is more important than in others (within optimum or extreme ranges) when predicting disease. This also suggests that relatively reliable models can be created for mosquito-borne diseases even if the high precision temperature data at extreme or optimum levels are not available.

In our current models, rainfall was consistently the weakest predictor variable. We attempted to improve the rainfall parameter by separating values into three categories as proxies for seasons, but the effects of rainfall on vector breeding and feeding behavior are in reality much more complex. For example, although rainfall generally increases the number of breeding places for mosquitoes, it fails to fully account for the most optimal breeding sites for *Ae. aegypti* (the vector for dengue), including man-made containers such as discarded tires or swimming pools. When containers are manually filled for water storage or recreation, mosquito abundance in urban areas can be largely independent of rainfall^38^. We used rainfall as an indicator for the amount of standing water, but to increase its predictive capabilities, the rainfall variable could be improved to include more detail about the intensity and frequency of rain each month.

There are many other important social and ecological factors involved in vector-borne disease seasonality that were not considered in this study. This limitation might in part explain the high residual skew (Figure S2 and Table S9), which indicates patterns in the disease time series beyond those accounted for in our models. Other environmental variables that could easily be incorporated into future versions of the models could include humidity or tidal data, both of which have been found to be significant indicators of vector-borne disease in Australia^30^,^50^. The high residual skew and kurtosis for the models also suggests that the models’ predictive accuracy, especially for dengue, is limited. This could be improved by using weekly rather than monthly case counts at a regional level, as the lower level of spatial aggregation would allow for a more nuanced model.

The models presented in Fig. 4 highlight the importance of modeling local and imported cases of dengue separately and reveal which factors can help predict dengue case counts based on place of acquisition. As one would expect, seasonality and climate are better indicators of disease rates for locally-acquired dengue than for overseas-acquired dengue. Since the data used in this study describe weather from Australia only, these parameters are expected to correlate with local dengue acquisition, and thus display more prominent multi-year periodicities than imported dengue. In contrast, the model for imported dengue instead relies heavily on the linear trend (Fig. 4c) meaning that Australians are more frequently contracting dengue while traveling abroad. This could mean that foreign travel is increasing, travel timing and destinations are shifting towards times and areas with higher dengue prevalence, or the countries which Australians visit are suffering from increased incidence of dengue. It is interesting to note that the estimated average peak timing for imported dengue is slightly earlier than that for locally-acquired dengue and overlaps with Christmas, a popular travel time in Australia.

An important limitation of this analysis is that it does not examine dengue importation between states within Australia. Although dengue is only endemic to QLD and possibly NT, travelers to these regions from other parts of Australia could be increasing reports of dengue throughout the states in Australia without endemic dengue. For this reason, this analysis was only able be performed on data from Australia as a whole and not for individual states. In order to better understand how imported and locally-acquired dengue affects each state independently, data on inter-state transmission of dengue must be reported. Better identification of location of exposure and a more accurate linkage of cases to vector habitat condition would lead to higher utilization of routinely collected data. In the study of hospitalizations due to respiratory infection in US, we had demonstrated that spatiotemporal patterns change due to population migration from northern to southern states during winter months, thus the seasonal patterns have to be properly adjusted^51^. Understanding the travel and seasonal migration is critical in preventing outbreaks. Unfortunately, publicly available data are typically crudely aggregated and thus, are unsuitable for a sophisticated analysis.

This study was the first to model and compare mosquito-borne diseases across Australia, with over twenty years of complete data on three common arboviral diseases. Using data from across an entire continent allows direct comparisons to be made between distant locations. By utilizing relatively few parameter variables and only publicly-sourced weather and case data, we attempted to create a generalized model that is widely accessible (all data used are free for public use) and adaptable (the same modelling approach can be used wherever similar data is available). While ground based meteorological monitoring might be limited in many resource-poor areas, the availability of remote sensing data is increasing. Further, our modeling approach successfully incorporated a Temperature Suitability Index that reduced the reliance on highly precise or localized temperature data, and we hope that this further increases the utility and applicability of the models to other large-scale geographic contexts. To improve these and similar models, the authors recommend that disease monitoring agencies provide case counts with smaller spatial and temporal aggregation and similarly provide numbers of imported versus locally-acquired cases. These actions will lead to an improved understanding of, and prediction accuracy for, vector-borne diseases, which account for nearly a fifth of all infectious diseases and millions of cases worldwide, and will enable health officials to be more prepared for periods of high disease incidence.

## Additional Information

**How to cite this article:** Stratton, M. D. *et al*. A comparative analysis of three vector-borne diseases across Australia using seasonal and meteorological models. *Sci. Rep.* **7**, 40186; doi: 10.1038/srep40186 (2017).

**Publisher's note:** Springer Nature remains neutral with regard to jurisdictional claims in published maps and institutional affiliations.

## Supplementary Material

Supplementary Materials

## Figures and Tables

**Table 1:** Creation of Model A, a basic model incorporating linear trend, seasonality and meteorological parameters.

Step	Model Content	Model Formulation
Step 1	Trend	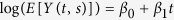
Step 2	Step 1 + Seasonality	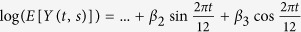
Step 3: Model A	Step 2 + Maximum temperature + Minimum temperature + Rain	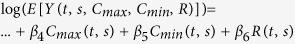

Variables: *t* = months since start of study (For January 1991, *t* = 1), *s* = state, *C* = average monthly temperature (maximum or minimum) in degrees Celsius, *R* = total monthly rainfall in millimeters.

**Table 2:** Creation of Model B, an improved model incorporating monthly lags and inter-annual periodicities.

Step	Model Content	Model Formulation
Step 1	Model A + Lag	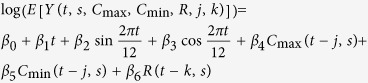
Step 2: Model B	Step 1 + Inter-annual Periodicities	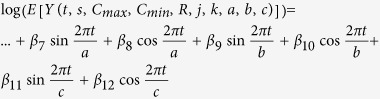

Variables: *t* = months since start of study (For January 1991, *t* = 1), *s* = state, *C* = average monthly temperature (maximum or minimum) in degrees Celsius, *R* = total monthly rainfall in millimeters, *j* = temperature lag in months, *k* = rain lag in months, *a, b, c* = inter-annual period lengths in months (Tables S6, S7).

**Table 3:** Creation of Model C.

Step	Model Content	Model Formulation
Step 1	Trend + Seasonality + Inter-annual Periodicities	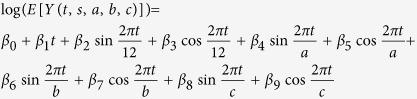
Step 2	Step 1 + TSI(Maximum temperature) + TSI(Minimum temperature) with lag	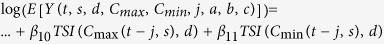
Step 3: Model C	Step 2 + Cool Rain + Warm Rain + Hot Rain with lag	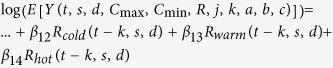

Variables: *t* = months since start of study (For January 1991, *t* = 1), *s* = state, *d* = disease, *C* = average monthly temperature (maximum or minimum) in degrees Celsius, *R* = total monthly rainfall in millimeters (during cold, warm, or hot months), *j* = temperature lag in months, *k* = rain lag in months, *a, b, c* = additional period lengths in months (Tables S6 and S7).
