# Supplementary material for: A comparative analysis of three vector-borne diseases across Australia using seasonal and meteorological models
Source: Sci Rep. 2017 Jan 10;7:40186. doi: 10.1038/srep40186 (PMC5223216; doi:10.1038/srep40186)
Supplement: Supplementary Materials [file srep40186-s1.pdf]

A comparative analysis of three vector-borne diseases across Australia using seasonal and meteorological models

Margaret D. Stratton; Hanna Y. Ehrlich; Siobhan M. Mor; Elena N. Naumova*

Supplementary Information

Table S1. Notifications of dengue infections, by arboviral season (July 1 – June 30), state, and place of acquisition. LA= locally-acquired*; OA = overseas-acquired; %OA = total percent acquired overseas**. All data abstracted from the Australian Department of Health's Annual Reports of the National Arbovirus and Malaria Advisory Committee, 2002-2013.

		2002-03	2003-04	2004-05	2005-06	2006-07	2007-08	2008-09	2009-10	2010-11	2011-12	2012-13	Total
**NSW/ACT**	**LA**	0	0	0	0	0	2	5	2	2	1	0	12
	**OA**	88	41	34	61	74	107	183	140	226	251	269	1474
	**(%)**	**100**	**100**	**100**	**100**	**100**	**98**	**97**	**97**	**95**	**97**	**96**	**97**
**NT**	**LA**	0	0	0	0	0	0	0	0	1	0	0	1
	**OA**	18	23	16	16	15	25	27	36	29	69	38	312
	**(%)**	**100**	**100**	**100**	**100**	**100**	**100**	**100**	**100**	**94**	**100**	**100**	**99**
**QLD**	**LA**	472	418	72	42	46	26	1003	33	125	16	206	2459
	**OA**	71	42	41	33	66	78	115	126	181	209	216	1178
	**(%)**	**13**	**9**	**36**	**44**	**59**	**72**	**10**	**79**	**59**	**93**	**51**	**32**
**SA**	**LA**	0	0	0	0	0	2	1	0	0	0	2	2459
	**OA**	8	7	3	10	12	31	26	11	28	44	47	1178
	**(%)**	**100**	**100**	**100**	**100**	**100**	**89**	**96**	**100**	**100**	**100**	**96**	**97**
**TAS**	**LA**	0	0	0	0	0	0	0	0	0	0	0	0
	**OA**	0	2	0	0	0	4	6	4	5	9	8	38
	**(%)**		**100**				**100**	**100**	**100**	**100**	**100**	**100**	**100**
**VIC**	**LA**	0	0	0	0	0	0	3	0	2	1	4	10
	**OA**	12	13	8	13	9	15	19	52	140	246	299	826
	**(%)**	**100**	**100**	**100**	**100**	**100**	**100**	**83**	**98**	**99**	**89**	**98**	**95**
**WA**	**LA**	0	0	2	0	0	0	0	0	1	0	0	3
	**OA**	21	6	11	20	27	94	121	226	525	561	325	1937
	**(%)**	**100**	**100**	**85**	**100**	**100**	**100**	**100**	**100**	**100**	**100**	**100**	**100**
**AUST**	**LA**	472	418	74	42	46	30	1012	35	131	18	212	2490
	**OA**	218	134	113	153	203	354	497	595	1134	1389	1202	5992
	**(%)**	**32**	**24**	**60**	**78**	**82**	**91**	**33**	**94**	**89**	**96**	**84**	**70**

*LA cases are acquired in Australia and not necessarily in the state or territory from which they are reported.
**%OA represents proportion of OA cases to total annual cases (as sum of LA, OA, and unknown place of acquisition cases). Cases with unknown place of acquisition were only provided for years 2007-8 through 2012-3 and were included only in these respective %OA calculations.

Table S2. Notifications of dengue infections, by year and country/place of acquisition. Data abstracted from Australia's Communicable Diseases Intelligence annual reports, with data corresponding to the report for its respective year, 1994-2014*. Empty cells represent no available data from reports.

Year	Locally-acquired (LA) cases	Overseas-acquired cases	Unknown place of acquisition	Total cases	Location of local acquisition	Surveillance notes
**1994**	0	17	0	17		1,056 cases reported in 1992-1993, primarily attributable to epidemic activity in QLD
**1995**				34		
**1996**				43		
**1997**				210	QLD reported over 80% of total cases	First case of 'airport malaria' (due to an aircraft-imported mosquito)
**1998**				557	QLD reported over 80% of total cases	
**1999**	45			131		NNDSS begins consistent data collection on importations
**2000**	11	153	51	215	All LA cases acquired in QLD	
**2001**	8	151	17	176	Highest no. of cases to-date in NSW (all NSW cases acquired overseas); all LA cases acquired in QLD	
**2002**	25			219	All LA cases acquired during 3 outbreaks in QLD	
**2003**				868	QLD reported 727 cases; all LA cases acquired in QLD	
**2004**	181	70	74	326	QLD reported 249 cases; all LA cases were acquired in QLD	First dengue fatalities (n=2) in over 100 years; incursion of *Ae. aegypti* into NT for first time since 1955
**2005**	74			218	QLD reported 115 cases; all LA cases were acquired in QLD	
**2006**				187	QLD reported 78 cases, 28 acquired locally	
**2007**	46	268		314	QLD reported 120 cases; all LA cases acquired in QLD	
**2008**	137	418	3	558	QLD reported 232 cases, most attributable to outbreak in N QLD	
**2009**	922	480	0	1,402	QLD reported 1,036 dengue counts; all LA cases acquired in outbreaks in N QLD	Largest number of LA cases ever reported
**2010**		1,119		1,201	9 LA cases reported in other states, but all LA cases acquired in N QLD	
**2011**	76	727	14	817	Most LA cases associated with 3 outbreaks in QLD; 1 healthcare-associated case from WA	98% completeness on place of acquisition
**2012**	29	1,410	101	1,540	28 LA cases associated with 5 epidemics in QLD	
**2013**	235	1,591	15	1,841	222 cases acquired from 10 epidemics in QLD; 1 case from WA	Change in CDNA case definition**; 99% completeness on place of acquisition; largest number of overseas acquisitions to-date
**2014**	186	1,520	10	1,716	All cases acquired in N QLD	99% completeness on place of acquisition

* CDI annual reports published annually 1994 through 2014.

** CDNA case definition for dengue was changed to accept dengue non-structural protein 1 (NS1) antigen in blood as laboratory definitive evidence for infection; however, a number of states had been sending notifications based on a positive NS1 antigen prior to this change.

Table S3. Notifications of dengue infections acquired overseas, by country of acquisition, 2009-2014. Total number of annual and overseas-acquired cases obtained from Australia's Communicable Diseases Intelligence annual reports, 2009-2014; data on selected countries of acquisition obtained from the 2014 report.

	**2009**	**2010**	**2011**	**2012**	**2013**	**2014**
Total cases	1,402	1,201	817	1,540	1,841	1,716
Imported cases	480	1,119	727	1,410	1,591	1,520
**Country of Acquisition***						
Indonesia	172 (36%)	717 (64%)	461 (63%)	804 (57%)	801 (50%)	811 (53%)
Thailand	25 (5%)	124 (11%)	85 (12%)	279 (20%)	269 (17%)	139 (9%)
Fiji	8 (2%)	1	6	32 (2%)	14	106 (7%)
Malaysia	16 (3%)	17 (2%)	21 (3%)	20	53 (3%)	81 (5%)
Philippines	9 (2%)	42 (4%)	24 (3%)	55 (4%)	63 (4%)	66 (4%)
Sri Lanka	0	4	12 (2%)	26 (2%)	28 (2%)	50 (3%)
India	15 (3%)	43 (4%)	31 (4%)	60 (4%)	58 (4%)	38 (2%)
Timor-Leste	25 (5%)	37 (3%)	12 (2%)	52 (4%)	49 (3%)	36 (2%)
Vanuatu	10 (2%)	4	0	0	5	33 (2%)
Singapore	1	4	5	3	18	15
Tonga	15 (3%)	1	0	0	0	15
Vietnam	20 (4%)	34 (3%)	14 (2%)	21	25 (2%)	15
Papua New Guinea	13 (3%)	21 (2%)	15 (2%)	16	35 (2%)	11
Nauru	0	0	0	0	0	11
French Polynesia	3	0	0	0	5	10
Cambodia	5	11	6	31 (2%)	31 (2%)	10

* percent of cases with reported country of acquisition based on the total number of imported cases (shown if greater than 1%)

Table S4. First- and second-order summary statistics for disease rates for each disease and state. Rates were measured in cases per million people. Disease data is aggregated by month in the range Jan 1995 – Oct 2012 for BFV, Jan 1993 – May 2015 for RRV, Jan 1991 – May 2015 for dengue, and July 2007 – June 2013 for local and imported dengue.

Disease	State	Mean	Median	Standard Deviation	Minimum	Maximum	Interquartile Range	Skew	Kurtosis
**BFV**	**AUST**	59.75	53.0	36.15	12	203	42.00	1.50	5.70
	**NSW**	60.68	47.0	49.40	4	266	44.75	1.92	6.82
	**NT**	233.90	172.5	234.21	0	1253	256.00	1.79	6.68
	**QLD**	165.87	145.0	101.37	31	778	102.25	2.23	11.06
	**SA**	21.60	8.0	42.26	0	278	29.00	3.79	19.92
	**VIC**	7.10	3.0	14.88	0	126	7.00	5.27	35.27
	**WA**	40.08	32.0	34.30	0	187	44.00	1.32	5.14
	**TAS**	1.49	0.0	5.83	0	25	0.00	3.66	14.43
**RRV**	**AUST**	230.06	150.0	231.45	15	1614	225.00	2.41	11.04
	**NSW**	139.20	83.0	151.99	5	931	134.00	2.10	8.09
	**NT**	1189.68	763.0	1352.47	0	9800	1237.00	2.71	13.32
	**QLD**	604.42	340.0	775.62	48	6401	534.00	3.54	20.11
	**SA**	156.80	63.0	357.45	0	3048	121.00	5.39	36.82
	**VIC**	62.49	20.0	154.12	0	1189	34.00	5.08	31.27
	**WA**	343.03	167.0	460.79	0	3107	349.00	3.01	14.46
	**TAS**	56.45	23.0	142.07	0	1345	49.00	5.28	38.52
**Dengue**	**AUST**	28.27	13.0	37.05	0	215	34.00	2.12	8.06
	**NSW**	32.04	11.0	41.78	0	221	46.00	1.73	6.00
	**NT**	131.49	63.0	210.65	0	1657	174.00	3.75	21.91
	**QLD**	66.50	20.0	136.35	0	1107	55.00	4.48	27.11
	**SA**	10.91	0.0	18.11	0	126	15.00	2.66	11.88
	**VIC**	11.48	0.0	23.42	0	138	7.00	2.52	9.29
	**WA**	53.61	7.0	100.01	0	546	49.00	2.57	9.64
	**TAS**	6.61	0.0	16.34	0	117	0.00	3.15	14.95
**Dengue (Local)**	**AUST**	0.91	0.0	2.64	0	15.94	0.53	4.36	22.01
**Dengue (Imported)**	**AUST**	48.72	31.72	40.09	6	199	53.91	1.59	5.56

Table S5. Coefficients for Model B*

Disease	State	β_0	β_1	β_2	β_3	β_4	β_5	β_6	β_7	β_8	β_9	β_{10}	β_{11}	β_{12}
**BFV**	**AUST**	0.96	4.09E-03	0.78	0.19	0.17	-0.10	-2.72E-03	0.05	0.03	0.02	0.07	0.09	0.09
	**NSW**	0.97	3.56E-03	0.74	0.09	0.18	-0.11	-2.08E-03	0.14	-0.01	0.06	0.14	0.09	0.09
	**NT**	-0.09	8.27E-03	0.31	0.59	0.10	0.05	-8.95E-04	0.03	0.18	0.13	0.43	0.09	0.25
	**QLD**	2.11	2.88E-03	0.61	0.29	0.09	0.02	-9.68E-04	0.05	0.01	0.03	-0.02	0.06	0.00
	**SA**	-2.94	2.40E-02	0.64	0.35	0.26	-0.43	6.41E-03	-0.08	0.14	0.29	0.95	0.23	1.29
	**VIC**	5.17	5.69E-03	2.08	-0.41	-0.27	0.04	9.71E-03	-0.04	0.31	0.16	-0.17	0.52	0.40
	**WA**	4.40	7.95E-03	0.40	-0.20	-0.11	0.04	3.34E-03	0.05	0.06	0.13	0.33	0.18	0.22
	**TAS**	-8.03	1.45E-02	1.64	-0.51	0.52	-0.42	-1.70E-02	0.33	0.62	0.79	0.67	-1.53	1.37
**RRV**	**AUST**	7.56	2.64E-04	1.25	0.07	-0.23	0.19	3.28E-03	0.05	-0.03	0.07	-0.01	0.03	0.15
	**NSW**	3.56	3.23E-04	-0.25	0.25	-0.20	0.40	2.33E-03	-0.01	0.07	0.08	-0.11	0.09	0.15
	**NT**	0.84	1.94E-04	1.32	0.40	0.14	0.08	-8.40E-04	0.12	0.06	-0.11	-0.02	0.17	-0.02
	**QLD**	5.01	-2.30E-03	0.64	-0.01	-0.02	0.10	1.67E-03	0.02	-0.13	0.12	0.09	0.01	0.21
	**SA**	5.18	3.56E-03	0.59	0.60	-0.44	0.67	1.05E-02	0.20	0.42	-0.15	-0.11	-0.07	0.16
	**VIC**	5.73	-4.79E-04	1.58	0.02	-0.37	0.42	1.16E-02	0.26	0.46	0.09	-0.14	-0.09	0.30
	**WA**	7.56	3.46E-03	1.26	0.25	-0.18	0.15	1.88E-04	-0.06	-0.30	0.44	-0.16	0.03	0.05
	**TAS**	6.03	-2.00E-03	2.13	-1.00	-0.24	0.12	6.93E-03	-0.18	-0.51	0.21	0.21	-0.57	-0.11
**Dengue**	**AUST**	2.39	8.01E-03	0.05	-0.29	-0.22	0.36	-4.42E-03	-0.28	0.23	-0.07	-0.30	0.39	0.20
	**NSW**	3.42	1.06E-02	0.29	0.25	-0.14	0.07	1.58E-03	-0.40	-0.01	-0.15	0.02	0.08	0.17
	**NT**	3.62	2.21E-03	0.20	-0.01	0.13	-0.17	4.61E-04	-0.59	0.27	0.14	-0.02	-0.12	-0.15
	**QLD**	11.38	3.37E-03	-0.10	2.50	-0.07	-0.38	-2.17E-03	-0.09	0.24	-0.18	-0.44	0.76	0.29
	**SA**	-2.82	1.43E-02	0.00	0.94	0.05	0.10	-6.14E-03	-0.28	0.03	0.25	0.12	-0.35	0.28
	**VIC**	-2.06	2.46E-02	0.39	0.24	-0.16	0.17	2.86E-03	-0.65	0.45	-0.14	-0.14	0.16	-0.10
	**WA**	-3.84	2.11E-02	-0.18	-0.53	0.07	0.08	2.74E-03	-0.75	-0.32	0.01	0.25	0.04	0.13
	**TAS**	-5.62	1.49E-02	-0.03	-0.86	0.36	-0.32	8.37E-03	-0.84	-0.04	0.25	-0.02	-0.39	0.80
**Dengue (Local)**	**AUST**	-16.7	7.37E-02	2.21	0.62	-0.09	-0.02	-4.96E-03	0.08	1.18	-0.47	0.16	0.69	2.97
**Dengue (Imported)**	**AUST**	338.6	-1.38	1.98	1.03	-0.15	-0.16	-3.66E-03	-29.1	45.29	3.21	0.15	6.71	14.05

*Model B:

$$\log(E[Y(t,s,C_{\max},C_{\min},R,j,k,a,b,c)]) = \beta_0 + \beta_1 t + \beta_2 \sin\frac{2\pi t}{12} + \beta_3 \cos\frac{2\pi t}{12} + \beta_4 C_{\max}(t-j,s) + \beta_5 C_{\min}(t-j,s) + \beta_6 R(t-k,s) + \beta_7 \sin\frac{2\pi t}{a} + \beta_8 \cos\frac{2\pi t}{a} + \beta_9 \sin\frac{2\pi t}{b} + \beta_{10} \cos\frac{2\pi t}{b} + \beta_{11} \sin\frac{2\pi t}{c} + \beta_{12} \cos\frac{2\pi t}{c}$$

Values for variables j, k, a, b, and c for each state and disease can be found in Table S6 and Table S7.

Table S6. Number of months of lag for temperature and rain variables for Models B and C. The lag used is the value between 0 and 12 that led to the model with the highest variation explained.

Disease	State	Model B Temperature Lag	Model B Rain Lag	Model C TSI Lag	Model C Rain Lag
BFV	AUST	7	6	9	11
	NSW	7	6	12	6
	NT	5	10	8	10
	QLD	6	6	0	6
	SA	12	5	6	11
	VIC	3	2	3	2
	WA	4	8	3	8
	TAS	9	7	6	7
RRV	AUST	3	2	8	2
	NSW	2	3	9	3
	NT	9	9	9	9
	QLD	2	2	8	2
	SA	2	5	5	5
	VIC	5	4	6	4
	WA	3	3	10	3
	TAS	4	5	4	5
Dengue	AUST	12	9	6	5
	NSW	12	6	9	6
	NT	7	12	11	12
	QLD	10	10	10	7
	SA	4	8	8	8
	VIC	1	11	12	1
	WA	12	0	12	1
	TAS	11	7	1	7
Dengue (Local)	AUST	2	9		
Dengue (Imported)	AUST	1	10		

Table S7. Inter-annual periodicities included in Models B and C.

Disease	Additional Periodicities
BFV	24, 60, 96 months
RRV	24, 36, 48 months
Dengue	48, 72, 144 months
Dengue (Local only / Imported only)	24, 36, 72 months

Table S8. Estimation of severity for major outbreaks in QLD, Australia from 1991-2009 based on modeling of state-wide cumulative case counts.

Year	Duration	Location	Serotype	Total observed cases	Total expected cases	Excess values	Severity index
1992-1993[52-55]	April 92 – July 93[52,53]	Townsville, Charters Towers[52]	Dengue 2 [52]	981	358	622	2.74
1996-1997[52,56-58]	Nov 96 – March 97[56]	Cairns[52]	Dengue 2 [52]	154	190	-36	0.81
1997-1999[59,60]	Dec 97 – March 99[52,60]	Cairns, Mossman, Port Douglas[52]	Dengue 2 & 3 [52]	559	365	194	1.53
2003-2004[61,62]	Jan 03 – April 04[61]	Cairns, Townsville[52]	Dengue 1 & 2 [67]	968	511	372	1.89
2008-2009[63-66]	Nov 08 – May 09[65]	Cairns, Townsville[52]	Dengue 1, 2, 3 & 4 [67]	1087	562	525	1.93

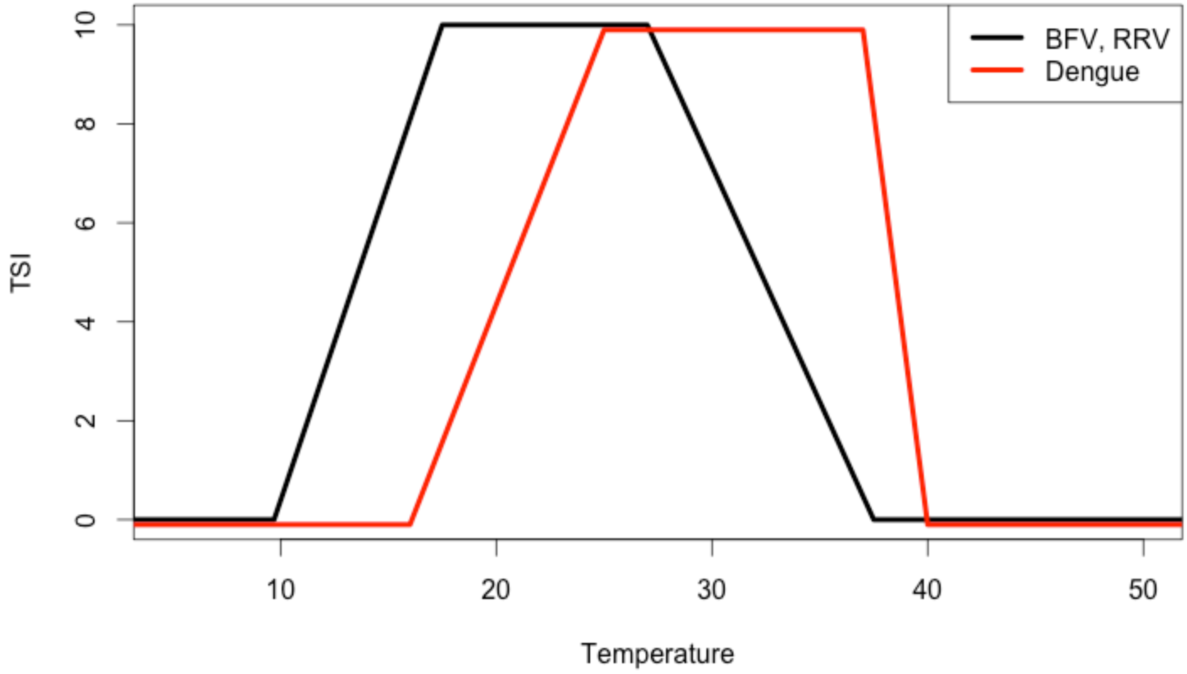

Figure S1. Calculation of Temperature Suitability Index: mapping from temperature in Celsius to TSI.

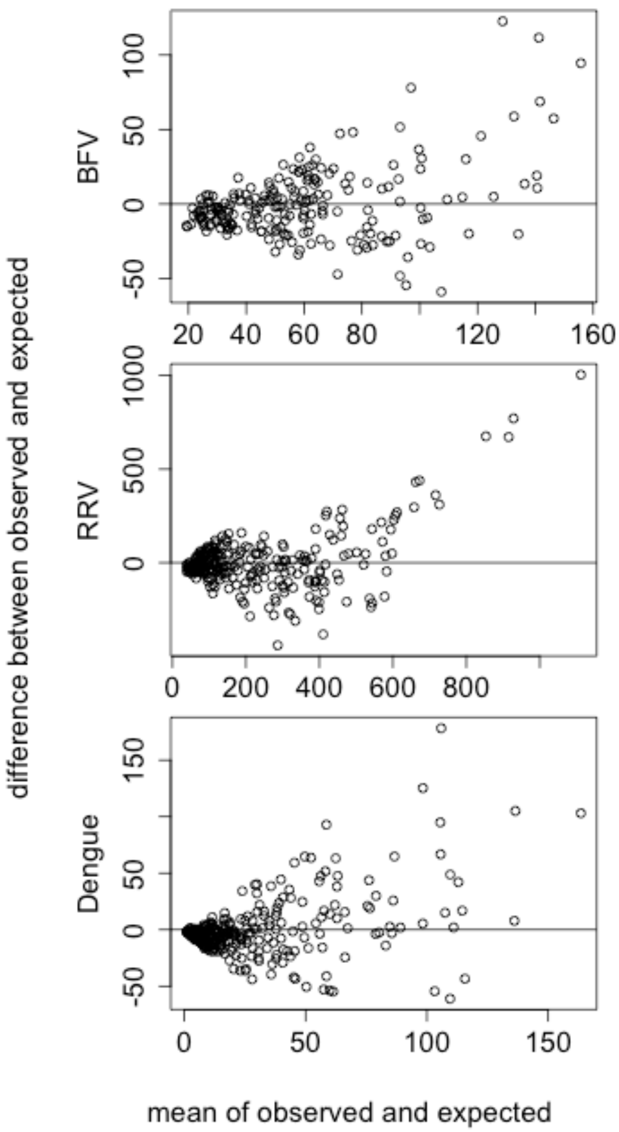

Figure S2. Graph of residual skew for Model B for BFV, RRV, and dengue across Australia.

Table S9. Measures of model fit for Model B for each state and disease.

Disease	State	Variability Explained	Residual Skew	Residual Kurtosis
BFV	AUST	0.65	1.24	5.11
	NSW	0.49	1.49	5.90
	NT	0.52	1.10	4.99
	QLD	0.49	1.62	7.43
	SA	0.78	6.05	47.33
	VIC	0.63	2.84	14.61
	WA	0.57	0.40	2.62
	TAS	0.37	6.42	51.76
RRV	AUST	0.69	0.98	3.61
	NSW	0.52	1.42	5.41
	NT	0.57	1.39	5.36
	QLD	0.62	2.06	7.88
	SA	0.54	5.01	42.65
	VIC	0.64	2.44	9.99
	WA	0.63	1.42	4.98
	TAS	0.64	3.72	20.20
Dengue	AUST	0.55	4.38	31.25
	NSW	0.59	2.21	10.61
	NT	0.44	2.39	12.41
	QLD	0.41	3.49	23.92
	SA	0.70	4.17	31.35
	VIC	0.88	8.07	73.99
	WA	0.87	4.54	33.14
	TAS	0.50	7.99	76.28
Dengue (Local)	AUST	0.91	3.20	14.05
Dengue (Imported)	AUST	0.63	1.19	4.45

Supplementary Works Cited:

52. Hanna, J.N. and Ritchie, S.A. Outbreaks of dengue in north Queensland, 1990–2008. Commun Dis Intell **33:1** (2009).
53. McBride, W.J.H., Mullner, H., LaBrooy, J.T. and Wronsk, I. The 1993 dengue 2 epidemic in Charters Towers, North Queensland: clinical features and public health impact. *Epidemiol Infect* **121**, 151–156 (1998).
54. Streatfield, R., Sinclair, D., Bielby, G., Sheridan, J., Pearce, M. and Phillips, D. Dengue serotype 2 epidemic, Townsville, 1992–93. Commun Dis Intell **17**, 330–332 (1993).
55. Murray-Smith, S., Weinstein, P. and Skelly, C. Field epidemiology of an outbreak of dengue fever in Charters Towers, Queensland: are insect screens protective? *Aust N Z J Public Health* **20:5**, 545-547 (1996).
56. Hanna, J.N., Ritchie, S.A., Merritt, A.D., van den Hurk, A.F., Phillips, D.A., Serafin, I.L., et al. Two contiguous outbreaks of dengue type 2 in north Queensland. *Med J Aust* **168:5**, 221–225 (1998).
57. McBride, W.J., Mullner, H., Muller, R., Labrooy, J. and Wronski, I. Determinants of dengue 2 infection among residents of Charters Towers, Queensland, Australia. *Am J Epidemiol* **148:11**, 111-6 (1998).
58. Ritchie, S.A., Hanna, J.N., Hills, S.L., Piispanen, J.P, McBride, W.J.H., Pyke, A. and Spark, R.L. Dengue control in north Queensland, Australia: case recognition and selective indoor residual spraying. *Dengue Bulletin* **26**, 7–13 (2002).
59. Hanna, J.N., Ritchie, S.A., Phillips, D.A., Serafin, I.L., Hills, S.L., van den Hurk, A.F., et al. An epidemic of dengue 3 in Far North Queensland, 1997–1999. *Med J Aust* **174:4**, 178–182 (2001).
60. Dengue 3 in Cairns: the story so far. *Commun Dis Intell* **22**, 109-110 (1998).
61. Hanna, J.N., Ritchie, S.A., Richards, A.R., Taylor, C.T., Pyke, A.T., Montgomery, B.L. et al. Multiple outbreaks of dengue serotype 2 in north Queensland, 2003/04. Aust N Z J Public Health **30:3**, 220-225 (2006).
62. McBride, W.J.H. Deaths associated with dengue haemorrhagic fever: the first in Australia in over a century. *Med J Aust* **183**, 35-37 (2005).
63. McCredie, J. Dengue fever epidemic hits northern Australia. *BMJ* **338:b967** (2009).
64. van den Hurk, A.F., Craig, S.B., Tulsiani, S.M. and Jansen, C.C. Emerging tropical diseases in Australia. Part 4. Mosquitoborne diseases. *Ann Trop Med Parasitol* **104:8**, 623-40 (2010).
65. Ritchie, S.A., Pyke, A.T., Hall-Mendelin, S., Day, A., Mores, C.N., Christofferson, R.C. et al. An Explosive Epidemic of DENV-3 in Cairns, Australia. *PLoS ONE* **8:7** (2013).
66. Hasan, T. and Bambrick, H. The effects of climate variables on the outbreak of dengue in Queensland 2008-2009. *Southeast Asian J Trop Med Public Health* **44:4**, 613-22 (2013).
67. Warrilow, D., Northill, J.A. and Pyke, A.T. Sources of dengue viruses imported into Queensland, Australia, 2002–2010. *Emerg Infect Dis* **18:11** (2012).
